# High-Throughput Empirical and Virtual Screening To Discover Novel Inhibitors of Polyploid Giant Cancer Cells in Breast Cancer

**DOI:** 10.1021/acs.analchem.4c05138

**Published:** 2025-03-05

**Authors:** Yushu Ma, Chien-Hung Shih, Jinxiong Cheng, Hsiao-Chun Chen, Li-Ju Wang, Yanhao Tan, Yuan Zhang, Daniel D. Brown, Steffi Oesterreich, Adrian V. Lee, Yu-Chiao Chiu, Yu-Chih Chen

**Affiliations:** † UPMC Hillman Cancer Center, University of Pittsburgh, 5115 Centre Ave, Pittsburgh, Pennsylvania 15232, United States; ‡ Department of Computational and Systems Biology, University of Pittsburgh, 3420 Forbes Avenue, Pittsburgh, Pennsylvania 15260, United States; § Department of Bioengineering, Swanson School of Engineering, University of Pittsburgh, 3700 O’Hara Street, Pittsburgh, Pennsylvania 15260, United States; ∥ Division of Malignant Hematology and Medical Oncology, Department of Medicine, University of Pittsburgh, 5150 Centre Avenue, Pittsburgh, Pennsylvania 15232, United States; ⊥ Department of Immunology, University of Pittsburgh, 5051 Centre Ave, Pittsburgh, Pennsylvania 15213, United States; # Institute for Precision Medicine, University of Pittsburgh, 5051 Centre Ave, Pittsburgh, Pennsylvania 15213, United States; ∇ Department of Pharmacology and Chemical Biology, University of Pittsburgh, 4200 Fifth Avenue, Pittsburgh, Pennsylvania 15213, United States; ○ CMU-Pitt Ph.D. Program in Computational Biology, University of Pittsburgh, 3420 Forbes Avenue, Pittsburgh, Pennsylvania 15260, United States

## Abstract

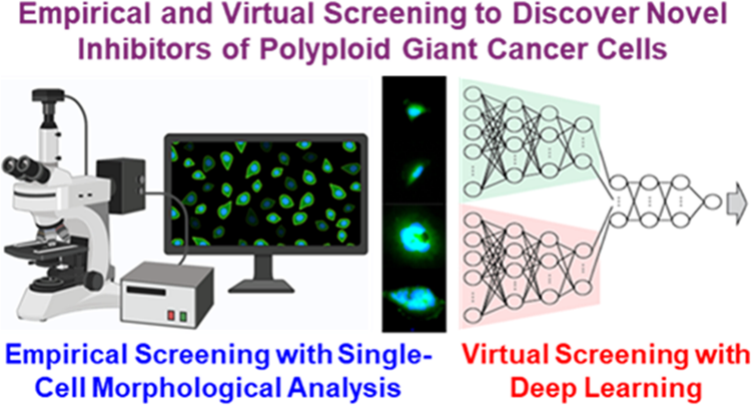

Therapy resistance in breast cancer is increasingly attributed to polyploid giant cancer cells (PGCCs), which arise through whole genome doubling and exhibit heightened resilience to standard treatments. Characterized by enlarged nuclei and increased DNA content, these cells tend to be dormant under therapeutic stress, driving disease relapse. Despite their critical role in resistance, strategies to effectively target PGCCs are limited, largely due to the lack of high-throughput methods for assessing their viability. Traditional assays lack the sensitivity needed to detect PGCC-specific elimination, prompting the development of novel approaches. To address this challenge, we developed a high-throughput single-cell morphological analysis workflow designed to differentiate compounds that selectively inhibit non-PGCCs, PGCCs, or both. Using this method, we screened a library of 2726 FDA Phase 1-approved drugs, identifying promising anti-PGCC candidates, including proteasome inhibitors, FOXM1, CHK, and macrocyclic lactones. Notably, RNA-Seq analysis of cells treated with the macrocyclic lactone Pyronaridine revealed AXL inhibition as a potential strategy for targeting PGCCs. Although our single-cell morphological analysis pipeline is powerful, empirical testing of all existing compounds is impractical and inefficient. To overcome this limitation, we trained a machine learning model to predict anti-PGCC efficacy *in silico*, integrating chemical fingerprints and compound descriptions from prior publications and databases. The model demonstrated a high correlation with experimental outcomes and predicted efficacious compounds in an expanded library of over 6,000 drugs. Among the top-ranked predictions, we experimentally validated five compounds as potent PGCC inhibitors using cell lines and patient-derived models. These findings underscore the synergistic potential of integrating high-throughput empirical screening with machine learning-based virtual screening to accelerate the discovery of novel therapies, particularly for targeting therapy-resistant PGCCs in breast cancer.

## Introduction

Polyploid giant cancer cells (PGCCs) are cancer cells with additional copies of chromosomes, often resulting in significantly larger cell size and increased genomic content.^1−3^ These cells are found across various cancer types, including breast, prostate, lung, ovarian, and colorectal cancers.^4−6^ The presence of PGCCs has been correlated to advanced disease stages, increased tumor aggressiveness, and poor clinical outcomes. The formation of PGCCs can be attributed to several mechanisms, including aberrant cell cycle regulation, mitotic failure, and response to cellular stress, such as chemotherapy and radiation. These mechanisms result in the cells bypassing normal mitotic checkpoints, leading to endoreduplication or cell fusion events that contribute to polyploidy.^7−9^ PGCCs contribute significantly to tumor heterogeneity. By reshuffling the genomic content of multiple copies of the genome,^10^ they generate diverse progeny through asymmetric division and budding, allowing for the rapid adaptation of tumor cells to changing microenvironments and therapeutic pressures.^11^ This adaptability promotes tumor evolution and metastasis, complicating treatment strategies.

PGCCs have emerged as a key target in cancer research due to their critical role in therapy resistance. These cells exhibit resistance to conventional chemotherapies and radiation therapy, often surviving initial treatments and giving rise to recurrent tumors.^9,12,13^ This resistance is mediated through multiple mechanisms, including enhanced DNA repair capabilities, activation of survival pathways, avoidance of apoptosis, and the ability to enter a dormant state. In addition, PGCCs are reported to exhibit stem cell-like properties by their enhanced tumor-initiating capability and upregulation of relevant biomarkers.^14−16^ Their presence often correlates with more aggressive disease phenotypes and poorer patient outcomes. Targeting PGCCs represents a promising therapeutic strategy. Approaches under investigation include disrupting the specific cell cycle and survival pathways active in PGCCs, as well as exploiting their unique metabolic dependencies.^17−20^ Therapies aimed at eliminating PGCCs or preventing their formation could enhance the treatment efficacy and reduce relapse rates.

Although there has been some progress in this direction, to date,^17−23^ there are no effective therapies targeting PGCCs.^9^ The development of anti-PGCC treatments has been hindered by the absence of a high-throughput method to rapidly quantify these cells. Traditional drug screening assays, such as MTT, XTT, or ATP, quickly measure the overall inhibition of cancer cell populations but fail to provide specific information on the elimination of a small PGCC subpopulation, which is crucial for addressing treatment resistance and relapse. PGCCs can be characterized by excessive DNA content and large cell and nuclear size. Currently, the gold standard for identifying and isolating PGCCs involves fluorescence-activated cell sorting (FACS) combined with visual confirmation.^14^ While flow cytometry can quantify the number and percentage of PGCCs, it is impractical for screening thousands of compounds or for monitoring the dynamic processes of PGCC induction and death. The limitations of existing approaches underscore the need for a high-throughput and precise analytical method specifically tailored to PGCC research.

Leveraging advancements in image-based cell segmentation and detection,^24−26^ we recently developed a dedicated single-cell morphological analysis pipeline to accelerate anti-PGCC therapy discovery.^27^ Using this pipeline, we developed complementary discovery strategies to identify novel PGCC inhibitors in this study: high-throughput screening of Phase 1-approved compounds for rapid translational impact, mechanistic studies to identify novel compound classes, and machine-learning-powered virtual screening to broaden the solution space. While our pipeline allows for high-throughput testing of thousands of compounds, exhaustive empirical testing of all existing compounds remains impractical, highlighting the critical role of computational methods in predicting anti-PGCC drug responses and prioritizing candidates for validation. Experimental screening generates essential data sets for building and evaluating various machine learning models, fostering a synergistic relationship between these approaches to streamline drug discovery.

**Figure 1:**
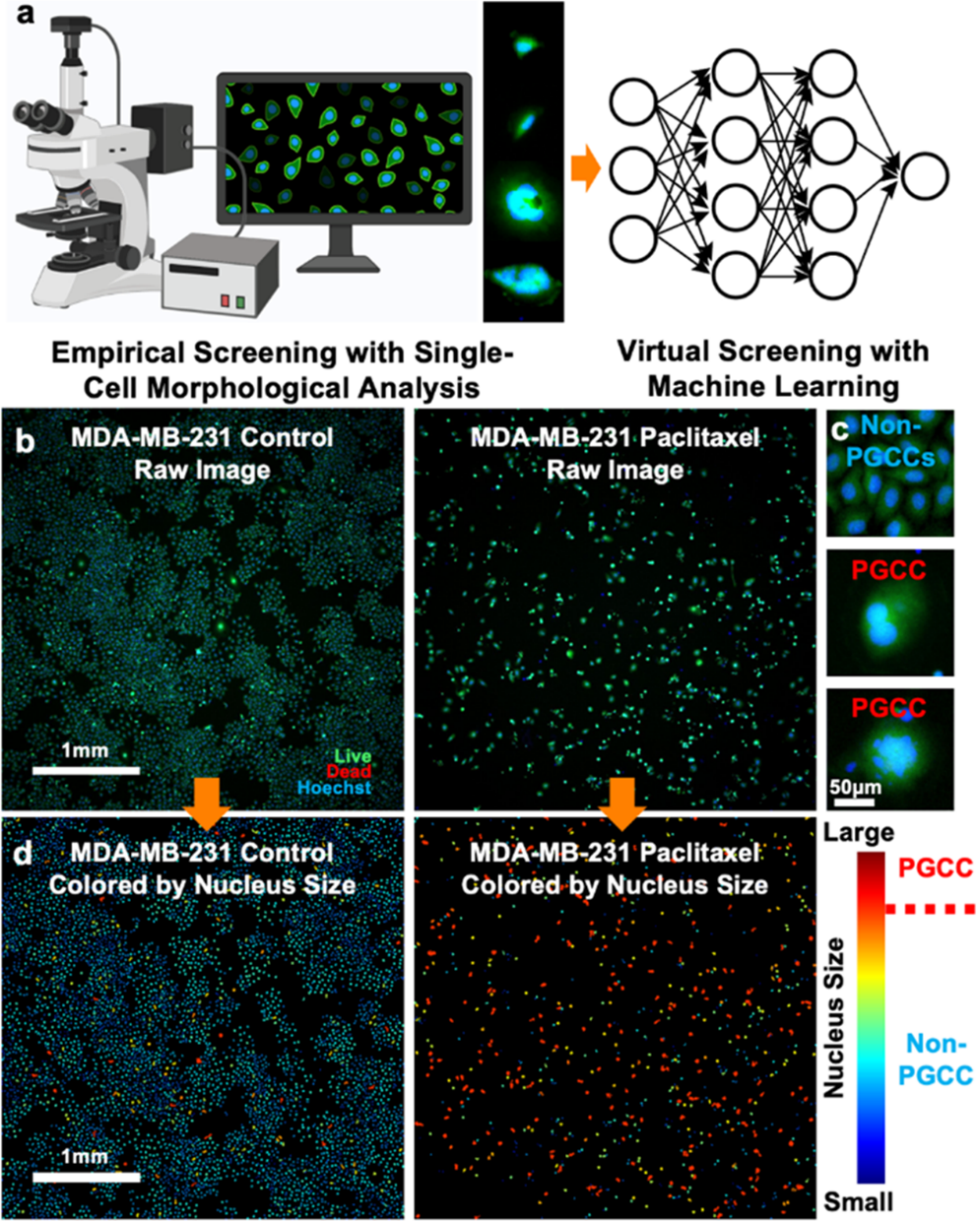
Single-cell morphological analysis for PGCC identification. (a) A conceptual diagram illustrating empirical drug screening by single-cell morphological analysis and virtual screening by machine learning. (b) Raw fluorescence images of MDA-MB-231 cells treated with or without Paclitaxel (Scale bar: 1 mm). Cells were stained with live (green), dead (red), and Hoechst (blue) reagents. (c) Enlarged images of representative MDA-MB-231 PGCCs and non-PGCCs (Scale bar: 50 μm). (d) Our single-cell morphological analysis pipeline converts raw images to pseudocolors indicating nuclear size: red for larger nuclei and blue for smaller nuclei.

Machine learning models have emerged as powerful tools, offering a promising solution by leveraging multiomics data and biochemical features of compounds, such as chemical structures, to predict drug sensitivity across cancer cell lines.^28−34^ However, to the best of our knowledge, no machine learning models currently exist for predicting anti-PGCC compounds, largely due to the lack of large training data sets. Establishing such methods is essential for advancing the development of targeted therapies for these challenging cancer cells. In this study, using our high-throughput morphological assay data, we developed an ensemble machine learning model integrating biochemical and pharmacological features to predict anti-PGCC activity (Figure 1a). Virtual screening of 6575 compounds identified top candidates, five of which were experimentally validated across four cell models. This study highlights the power of AI-driven and empirical screening to accelerate PGCC inhibitor discovery and combat therapy resistance.

## Methods

### Single-Cell Morphological Analysis to Identify Inhibitors of PGCCs

In our screening experiments, we utilized a compound library of 2726 compounds, each having successfully completed Phase I drug safety confirmation (APExBIO, L1052, DiscoveryProbe Clinical & FDA-Approved Drug Library). These compounds were prepared at a concentration of 10 mM in DMSO or PBS and diluted to a final concentration of 10 μM for screening. Cells were harvested from culture dishes using 0.05% Trypsin/EDTA (Gibco, 25,200), centrifuged at 1000 rpm for 4 min, resuspended in appropriate media, and seeded into 96-well plates. For direct treatment, 1000 cells were seeded in 100 μL of media per well. Cells were cultured for 24 h before treatment with compounds for 48 h. Post-treatment, cells were stained with 0.3 μM Calcein AM (Biotium, 80011–2), 0.6 μM ethidium homodimer-1 (Invitrogen, L3224), and 8 μM Hoechst 33342 (Thermo Scientific 62249), followed by a 30 min incubation. For preinducing PGCC experiments, 4000 cells per well were seeded. After 24 h, cells were treated with a PGCC-inducing agent (Docetaxel 1 μM) for 48 h. Postinduction, the reagents were aspirated, and the test compounds were added to treat the mixed populations for an additional 48 h. The same staining and imaging protocol was used to quantify PGCCs and non-PGCCs after treatments. Loading cells, drugs, or staining reagents into a 96-well plate requires less than 10 s with our pipetting robot, accommodating 88 test conditions and 8 control wells for normalization. To quantify PGCCs and non-PGCCs in collected images, we developed a custom MATLAB (2022b) program to achieve this in three steps: (1) identify cell nuclei with Hoechst staining, (2) determine cell viability, and (3) recognize PGCCs based on nuclear size based on our previous work.^27,35−37^ Among the 2726 compounds, 29 compounds were excluded due to their fluorescent colors, which interfere with image processing.

### Representation of Drug Features Using Structures and Descriptions

For machine learning modeling, each drug was represented by either a vector of molecular fingerprints to capture its biochemical and structural features or a vector of text embeddings to encode descriptions of its pharmacological, biochemical, and molecular biological properties. Drug structures were represented by the Simplified Molecular Input Line Entry System (SMILES) line notation. Canonical SMILES codes were obtained from PubChem using the Python PubChemPy package and then converted into molecular fingerprints based on the Molecular ACCess System (MACCS), PubChem, and Extended-Connectivity Fingerprint (ECFP6) systems using the R rcdk package.^38^ The molecular fingerprints are binary vectors that encode the structural properties of a drug, with lengths of 166, 881, and 1024 bits, respectively, where each bit denotes the presence (1) or absence (0) of a predefined structural property. Text descriptions of drugs were obtained from PubChem using the PUG REST interface, which provides programmatic access to PubChem data.^39,40^ We then converted the descriptions into text embeddings using the latest embedding methods developed by OpenAI, including text-embedding-3-small (1536 dimensions) and text-embedding-3-large (3072 dimensions), which generate vectors composed of continuous values to represent the semantic information on drug descriptions.

### Machine Learning Models to Predict Anti-PGCC Efficacy

We trained machine learning models to predict drug responses in PGCCs of MDA-MB-231 based on drug structures and descriptions. The normalized count of PGCCs, compared between treated and untreated cells, was increased by 10^–3^ and then log 2-transformed and used as the prediction target. We employed 10-fold cross-validation to train and test each model. In each round of 10-fold cross-validation, the drugs were randomly partitioned into 10 sets, where 9 sets were used for model training, and the remaining set was used for testing, where a Pearson correlation coefficient was calculated between the actual and predicted values. Once all 10 sets were tested by the corresponding trained models, we summarized the performance by averaging the 10 correlation coefficients. This entire process, including random partitioning and a 10-fold cross-validation, was repeated for 10 rounds. The results from these 10 rounds were presented in box plots, with performance summarized by the median correlation value. We evaluated a total of seven linear and nonlinear regression-based machine learning models, including linear regression with L2 regularization (Ridge), support vector machine (SVM), random forest (RF), histogram-based gradient boosting (HGB), decision tree (DT), stochastic gradient descent linear regression (SGD), and multilayer perceptron (MLP). These models were implemented by using the respective functions of the Python scikit-learn library. For ensemble learning, the predicted drug responses from two individual models, trained on either drug structures or descriptions, were used as inputs for training a linear regression model to predict the drug response. We ensured that all random partitions were applied consistently across individual and ensemble models to allow for a rigorous comparison of the results.

## Results and Discussion

### Comprehensive Compound Efficacy Analysis by Quantifying PGCCs and Non-PGCCs

We developed a high-throughput single-cell morphological analysis pipeline to quantify PGCCs and non-PGCCs by segmenting nuclei with Hoechst staining, excluding dead cells via Live/Dead staining, and classifying cells based on nuclear size (Figure 1a).^27^ Validated across multiple breast cancer cell lines, our approach aligns with flow cytometry and manual inspection.^27^ As a demonstration, Paclitaxel treatment of MDA-MB-231 cells significantly reduced the total cell count while enriching PGCCs (Figure 1b–d). Our image-processing pipeline converts raw images into pseudocolored representations, revealing a clear shift toward larger nuclei (red) in treated cells, confirming PGCC induction. Leveraging this pipeline, we screened a library of 2726 Phase I-approved compounds for their impact on PGCCs and non-PGCCs. Among 2726 compounds, 29 fluorescent-interfering compounds were excluded, and 461 inhibited the total cell number by at least half. However, among those 461 compounds, 236 compounds (51.2%) enriched PGCCs by at least 2-fold. Notably, standard chemotherapies, including Taxanes, Gemcitabine, Carboplatin, and Vinorelbine, depleted non-PGCCs but expanded PGCC populations, explaining tumor resistance and relapse post-treatment. In contrast, Cyclophosphamide, Capecitabine, and Fluorouracil did not induce PGCCs but showed limited efficacy in cancer cell elimination. These findings underscore the limitations of current triple-negative breast cancer (TNBC) therapies and highlight the necessity of PGCC-targeting strategies, for which our screening pipeline provides a powerful discovery platform.

### Discovering PGCC Inhibitors with Screening Experiments

**Figure 2:**
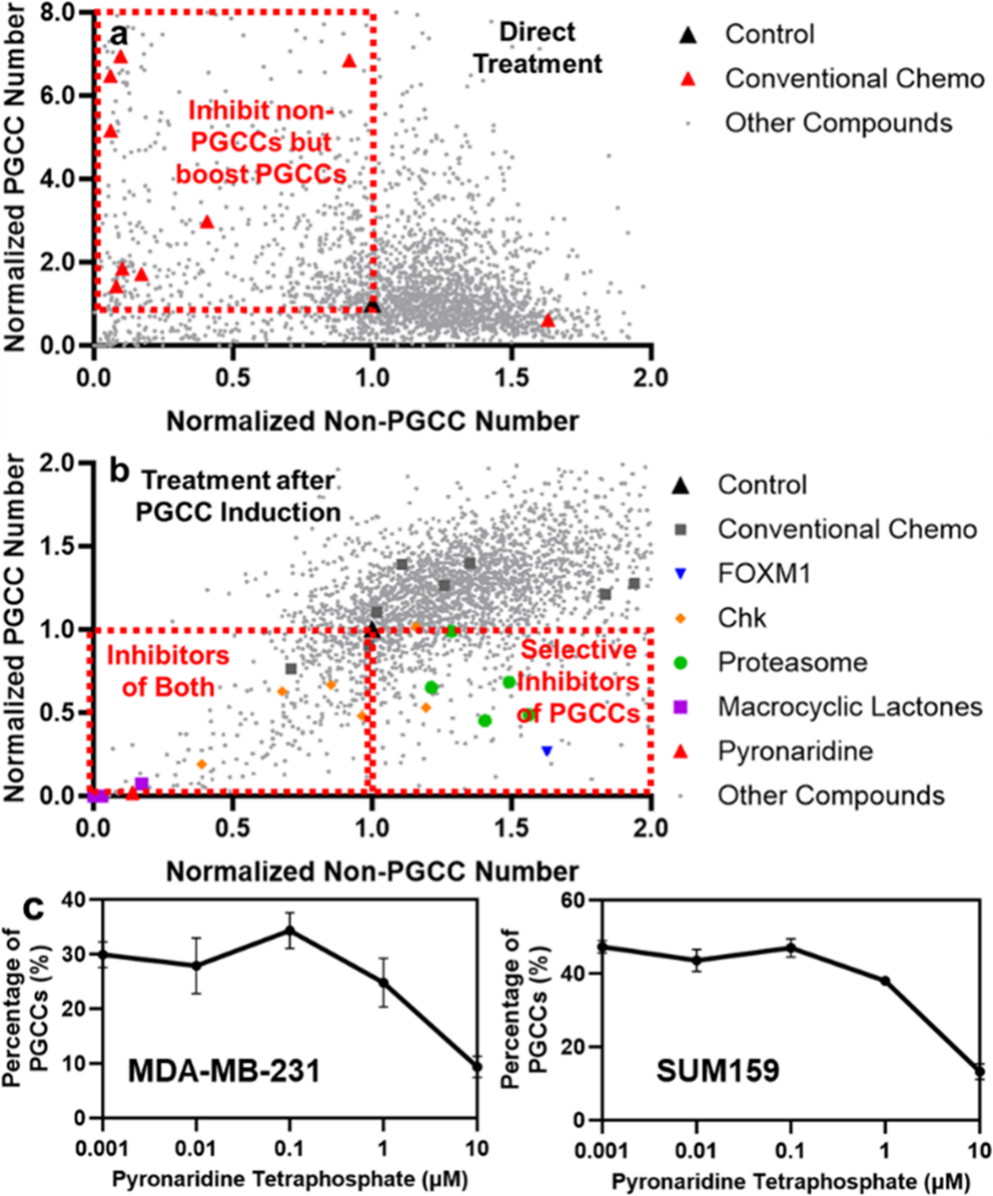
Screening of compounds using MDA-MB-231 breast cancer cells. The *X*-axis represents the number of non-PGCCs after treatment, and the *Y*-axis represents the number of PGCCs. Each dot represents the effect of a compound. (a) Direct treatment with screening compounds. (b) Pretreatment with Paclitaxel to induce PGCCs before drug screening. (c) Pyronaridine treatment effects on TNBC cell lines. Error bars: SD; *n* = 4.

Since most TNBC cell lines naturally contain fewer than 1% PGCCs, evaluating compound efficacy against PGCCs is challenging. To enrich PGCCs, we pretreated cells with Docetaxel for 2 days before introducing test compounds for an additional 2 days, followed by staining and imaging (Figure 1a). As shown in Figure 2b, drug-resistant PGCCs remained resistant to most chemotherapeutics. Among 2697 screened compounds, 169 reduced PGCCs by at least 2-fold, 45 by 10-fold, and 63 inhibited both PGCCs and non-PGCCs by at least 2-fold (Figure 2b). Notably, proteasome inhibitors (Bortezomib, Oprozomib, Carfilzomib, Celastrol), CHK inhibitors (AZD7762, PF-477736), and the FOXM1 inhibitor Thiostrepton emerged as potent PGCC-targeting agents. FOXM1, a key cell cycle regulator, is dysregulated in PGCCs, making them particularly vulnerable to its inhibition.^27,41,42^ Proteasome inhibitors induce cell death through multiple mechanisms, including pro-apoptotic protein accumulation, cell cycle arrest, and heightened sensitivity to other therapies.^43,44^ CHK inhibitors, by targeting CHK1/CHK2, impair DNA damage repair and cell cycle control, enhancing therapy-induced cancer cell death.^45,46^ While these compounds are well studied, they are not yet clinically used for breast cancer treatment resistance. Their selective activity against PGCCs underscores their potential as targeted therapies to overcome treatment resistance.

In addition, our large-scale screening identified novel PGCC-targeting compounds beyond the well-characterized drug classes (Figure 2b). Notably, macrocyclic lactones, including Doramectin, Pyronaridine, Ivermectin, and Moxidectin, known for their antiparasitic activity,^47,48^ disrupt neurotransmission by modulating glutamate-gated chloride channels, selectively affecting parasites while sparing host cells. While Doramectin has been shown to inhibit glioblastoma cell survival via autophagy modulation,^49^ its role in breast cancer remains unexplored. Additionally, Pyronaridine, an antimalarial drug,^50,51^ emerged as a potent PGCC inhibitor. It disrupts hemozoin formation, intercalates DNA, and induces oxidative stress, leading to parasite death. Pyronaridine also exhibits antiviral activity against COVID-19 and Ebola.^52,53^ Although its potential impact on breast cancer has been noted,^54,55^ there has been no prior investigation into its potential in targeting cancer resistance and PGCCs. While the precise mechanisms underlying PGCC inhibition remain unclear, these compounds offer promising avenues for future research. To validate our findings, we further validated it with multiple concentrations and cell lines (Figure 2c). Pyronaridine selectively eliminated PGCCs in both models, highlighting our ability to identify new compounds with PGCC-specific activity.

### Identification and Validation of AXL as a Key Mediator for the Anti-PGCC Effects of Pyronaridine

**Figure 3:**
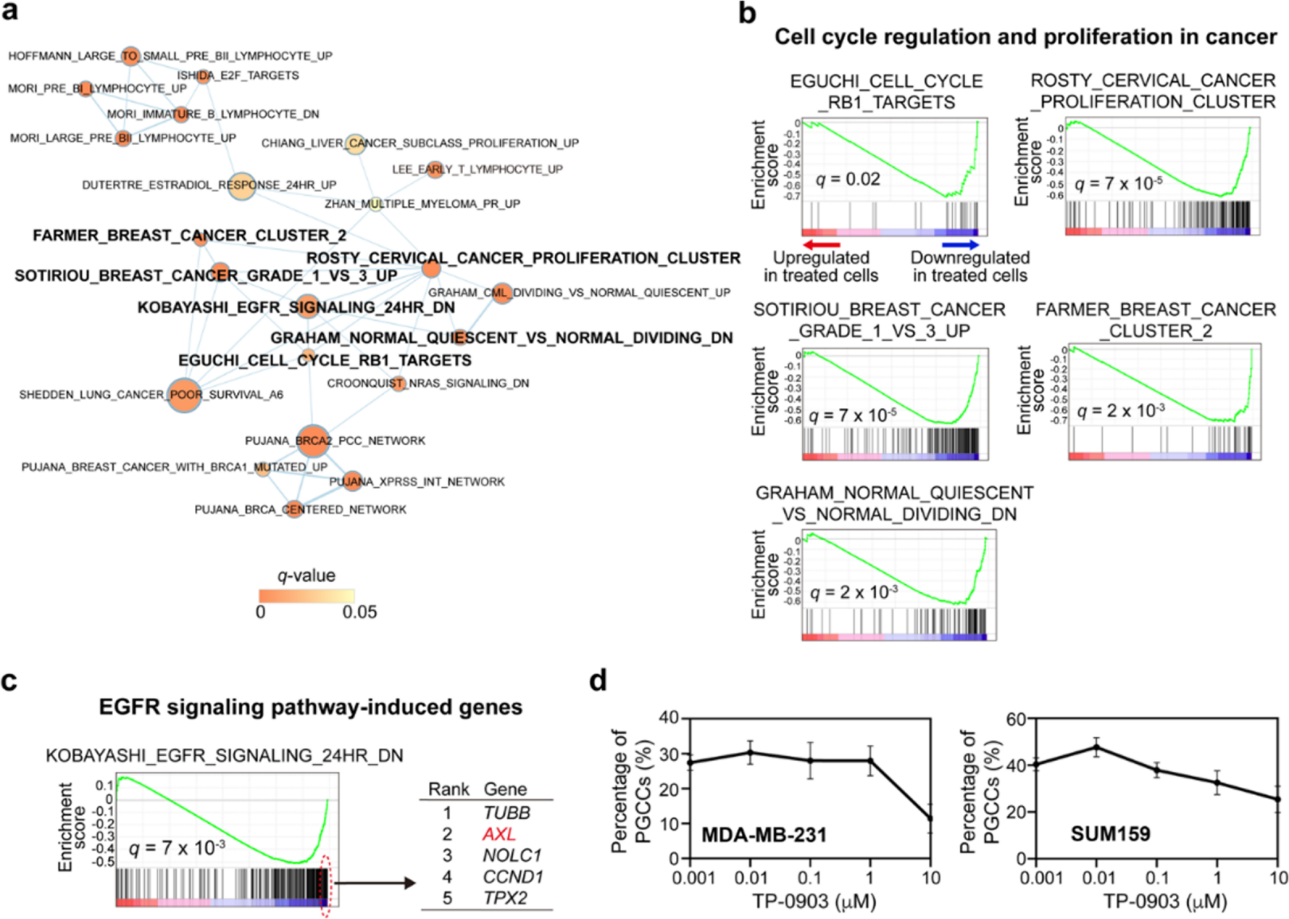
RNA-Seq analysis of Pyronaridine treatment and validation of TP-0903, an AXL inhibitor, in inhibiting PGCCs. (a) GSEA of Pyronaridine-treated cells compared to untreated cells was performed using curated CGP gene sets of MSigDB. A gene set association network was constructed among the significantly depleted gene sets and visualized by EnrichmentMap. Each node represents each gene set (node size: gene set size; node color: GSEA *q*-value; edge width: degree of gene overlap between two gene sets [combined coefficient >0.375]). Gene sets highlighted in bold are further shown in the following panels. (b, c) Significantly depleted gene sets associated with cell cycle regulation and proliferation (b) and EGFR signaling pathway (c) in Pyronaridine-treated cells compared to untreated cells. The five top-ranked leading-edge genes in the EGFR signaling pathway gene set are shown (c, right panel). (d) Effects of TP-0903 on two TNBC cell lines. Error bars: SD; *n* = 4.

To elucidate the mechanisms underlying Pyronaridine’s inhibition of PGCCs in MDA-MB-231 cells, we performed RNA-seq on Pyronaridine-treated PGCCs and compared their gene expression profiles to untreated controls. GSEA identified 283 significantly depleted gene sets enriched for genes downregulated by Pyronaridine. Network analysis revealed a strong association with cell cycle regulation and cancer proliferation (Figure 3a,b). Among these gene sets, the KOBAYASHI_EGFR_SIGNALING_24HR_DN gene set, linked to EGFR inhibition, was significantly depleted (NES = −1.74, *q* = 0.007) (Figure 3a–c).^56^ This set overlapped with others related to cell cycle states, RB1 targets, and breast cancer grades, suggesting that Pyronaridine disrupts EGFR signaling to inhibit PGCC proliferation in TNBC. These findings align with prior reports of Pyronaridine’s effects in non-small cell lung cancer.^57^

We further explored key players in the EGFR signaling pathway-mediated genes for their potential as therapeutic targets of PGCCs in TNBC. The top five leading-edge genes from GSEA (TUBB, AXL, NOLC1, CCND1, and TPX2) were all significantly downregulated by Pyronaridine (Figure 3c). Among them, AXL emerged as a particularly promising target. AXL, a receptor tyrosine kinase, regulates cell survival, proliferation, migration, and invasion.^58−60^ In PGCCs, AXL may drive DNA damage response and cytokinesis failure,^61,62^ thereby supporting the growth and adaptation of polyploid cancer cells under stressed conditions. Given our RNA-Seq data and its potential role in therapy resistance, we tested TP-0903, a novel ATP-competitive AXL inhibitor, in clinical trials for advanced solid tumors.^63,64^ TP-0903 effectively eliminated PGCCs in both the MDA-MB-231 and SUM159 cells (Figure 3d). This preliminary study aligns with RNA-Seq analysis and supports that Pyronaridine’s mechanism in targeting PGCCs may involve the AXL pathway.

### Machine Learning-Based Prediction of Anti-PGCC Effects

**Figure 4:**
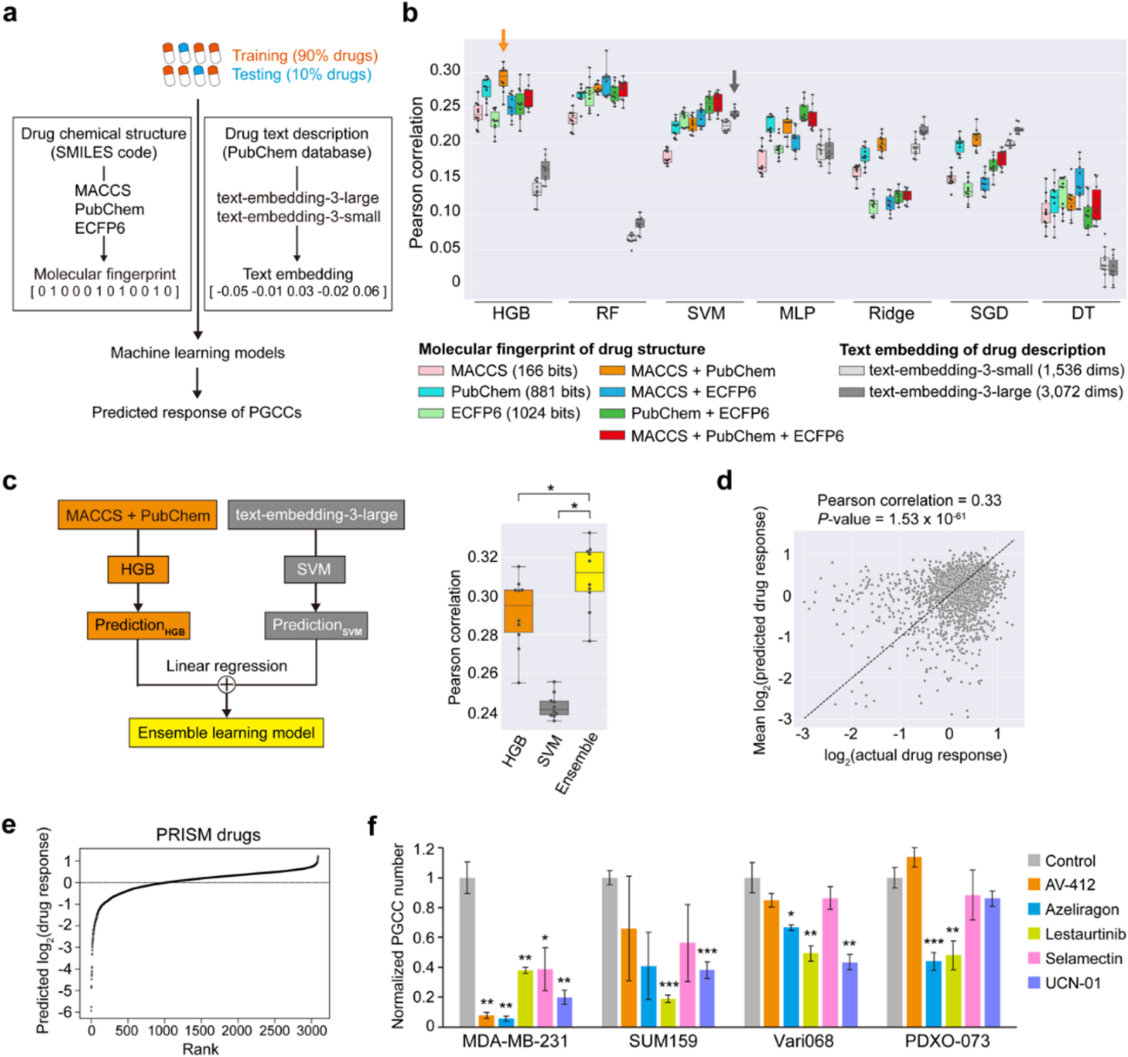
Machine learning framework for predicting PGCC drug responses. (a) We trained machine learning models to predict PGCC drug responses using drug chemical structures (fingerprints) and text descriptions (embeddings). A total of 2430 compounds were included, with 2187 for training and 243 for testing. (b) Seven state-of-the-art models were evaluated for comparison. Performance, measured by Pearson correlation, was assessed for single and multifingerprint models as well as text embeddings. Each dot in the box plots represents the average of 10 correlation coefficients obtained from 10-fold cross-validations in each round. Orange and gray arrows highlight the best models selected for ensemble learning. (c) Left panel: schematic of an ensemble learning model trained by integrating the best-performing models for drug structures and descriptions. Right panel: predictive performance comparison of the ensemble learning model versus individual models (*: one-tailed paired *t*-test *P* < 1 × 10^–6^). (d) Predictive performance of the ensemble model across all 2430 drugs. For each drug, the average predicted response across the 10 rounds is shown in the plot. Only drugs with log_2_-transformed response values > −3 are shown. (e) The rank plot of predicted log_2_-transformed drug response values in PGCCs of MDA-MB-231 cells, using our ensemble learning model based on PRISM project drugs. (f) Experimental validation of five predicted compounds in 2 TNBC cell lines (MDA-MB-231, SUM159) and 2 low-passage, patient-derived breast cancer models (Vari068, PDXO-073). PGCC counts were normalized to control. Error bars: SEM, *P < 0.05, **P < 0.01, ***P < 0.001 (compared with control). Detailed results in Table S1.

Although our assay enables high-throughput compound screening, empirically evaluating all existing compounds is neither practical nor efficient. To overcome this limitation, we developed predictive machine learning models trained on our experimental data. To the best of our knowledge, this is the first study to apply machine learning to predicting the anti-PGCC efficacy of compounds. We systematically evaluated seven state-of-the-art regression models to predict the PGCC-targeting effects in MDA-MB-231 cells. These regression models were trained to predict changes in PGCC counts based on quantitative representations of either chemical structures (fingerprints) or compound descriptions (text converted to embeddings) (Figure 4a). To maximize predictive power, we generated fingerprints using three complementary widely used descriptor systems (MACCS, PubChem, and ECFP6), capturing key structural and connectivity-based features. For text-based embeddings, we utilized drug descriptions from PubChem, integrating data from multiple well-established databases, including DrugBank, ChEBI, NCIt, MeSH, and Open Targets. This comprehensive approach mitigates biases from any single database and enhances the robustness of our predictive models by incorporating chemical, pharmacological, and clinical insights.

A total of 2430 compounds in the screening library with both features available were used in the model. We adopted 10 rounds of 10-fold cross-validations to train and test each model. In each iteration of cross-validation, a model was trained using 90% of the 2430 compounds and tested on the remaining 10%, which were not seen by the model during training. Overall, 31 out of 63 (49.2%) models achieved a median Pearson correlation coefficient ρ above 0.2 across 10 rounds of cross-validations (Figure 4b).

For molecular fingerprints, HGB with a combination of MACCS and PubChem was the best model (median ρ, 0.29; Figure 4b). Models that used combinations of multiple molecular fingerprints as features tended to achieve better performance compared with those using single molecular fingerprints. For example, HGB with MACCS and PubChem, RF with MACCS and ECFP6, and SVM with all three molecular fingerprints outperformed their single-fingerprint counterparts (Figure 4b). For description-based embeddings, models with longer embeddings (3072 dimensions) generally outperformed those with 1536 dimensions (Figure 4b), suggesting that longer embeddings capture additional pharmacological information. Notably, SVM with 3072-dimensional embeddings was the best-performing model (median ρ = 0.24; Figure 4b). Overall, the performance of these models was comparable to the best results from a community challenge for predicting drug sensitivities and recent studies predicting genetic dependencies in pan-cancer cell lines,^65−67^ demonstrating the capability of our screening library to support accurate predictive modeling.

### Enhancing Predictive Performance by Integrating Compound Structures and Descriptions Using an Ensemble Learning Approach

Since compound structures and descriptions provide distinct yet potentially complementary information, combining these features may improve the performance of predictive models. To explore this, we developed an ensemble learning method by integrating the best-performing models for drug structures and descriptions (*i*.*e*., HGB on MACCS and PubChem and SVM on the longer embedding). The ensemble model utilized linear regression to generate the final prediction based on the outputs of these two models. Notably, this approach significantly improved performance (median ρ = 0.31) compared to the individual models (one-tailed paired *t*-test, both *P* < 1 × 10^–6^) (Figure 4c). Across all 2,430 drugs, the ensemble model achieved a ρ of 0.33 between actual and predicted drug responses (*P* = 1.53 × 10^–61^) (Figure 4d).

In the ensemble model, the regression coefficients for the HGB and SVM models were 1.2 and 0.6, respectively, both statistically significant (*P* < 1 × 10^–3^). These results suggest that both models contributed meaningful and independent information to the ensemble model. The HGB model had a greater impact on the final prediction, while the SVM model predictions provided a complementary effect. Taken together, our findings demonstrate that integrating these two distinct features allows the model to capture meaningful and complementary patterns related to anti-PGCC effects, leading to enhanced predictive performance.

### Expanded Virtual Screening by the Ensemble Prediction Model and Experimental Validation

We expanded our virtual screening to a broader range of compounds to identify potential anti-PGCC agents in breast cancer. As a proof of concept, we compiled a large library of compounds based on the Profiling Relative Inhibition Simultaneously in Mixtures (PRISM) project, which is one of the largest drug sensitivity screens, covering 6575 oncology or non-oncology drugs (as of 24Q2).^68^ Of these 6575 drugs, 3093 drugs were not included in our original screening library but had both drug structure and description information. We applied our ensemble model to predict anti-PGCC effects for these 3093 drugs in MDA-MB-231 cells. The predicted drugs are ranked based on their inhibition effects in PGCCs (Figure 4e). Among the top-ranked candidates, we prioritized five compounds, Selamectin, AV-412, Azeliragon, Lestaurtinib, and UCN-01, based on novelty, pharmacological strength, and translational potential for experimental validation. All five compounds effectively inhibited PGCCs in MDA-MB-231 cells, confirming the model’s predictive power (Figure 4f and Table S1).

To enhance clinical relevance, we further validated these compounds in an additional TNBC cell line (SUM159) and two patient-derived breast cancer models, Vari068 (TNBC) and PDXO-073 (ER+).^69−71^ Lestaurtinib consistently suppressed PGCCs across all models, while UCN-01 and Azeliragon exhibited efficacy in three models. In contrast, Selamectin and AV-412 showed limited activity beyond MDA-MB-231, suggesting intercellular variability (Figure 4f and Table S1). These results highlight the influence of cell line-specific differences. Future work will incorporate additional training data from diverse cell models and integrate molecular features (*e*.*g*., key mutations) to refine predictive accuracy and enhance clinical translation.

While machine learning models cannot provide mechanistic explanations, a literature review underscores the therapeutic potential of the identified compounds. Lestaurtinib, a targeted FLT3 inhibitor, disrupts stress signaling pathways like JAK2 and has shown promise in relapsed FLT3 mutant acute myeloid leukemia,^72,73^ with potential applications in solid tumors warranting further exploration. UCN-01 (7-hydroxystaurosporine), a Chk1 inhibitor, disrupts critical cell cycle checkpoints, targeting PGCCs’ ability to manage DNA damage and genomic instability.^74,75^ It has exhibited encouraging results in early-phase cancer trials with ongoing efforts to optimize its pharmacokinetics and reduce plasma protein binding. Modifications to enhance its drug-like properties could unlock its potential for broader clinical application. Azeliragon, a RAGE inhibitor, has shown versatility with ongoing trials exploring its therapeutic potential beyond Alzheimer’s disease.^76,77^ Current investigations, including a Phase II trial in glioblastoma, underscore its potential to address critical challenges in cancer treatment, including therapy-related toxicity and tumor microenvironment modulation. Selamectin, FDA-approved for veterinary use, presents a promising starting point for cancer therapy due to its established safety and dosing in animals.^78^ With focused preclinical studies addressing pharmacokinetics, tissue-specific toxicity, and human ion channel interactions, Selamectin could potentially be repurposed for cancer treatment,^79^ offering a novel therapeutic avenue. AV-412, an oral tyrosine kinase inhibitor targeting EGFR and HER2, has demonstrated preclinical efficacy, including activity against resistant tumor models.^80,81^ Its successful completion of a Phase I trial highlights its potential for further development, paving the way for additional studies to establish its clinical utility in cancer therapy. Overall, our investigation demonstrates the significant potential of machine learning-based virtual screening to accelerate the discovery of novel anticancer therapies, particularly for targeting therapy-resistant PGCCs.

## Conclusions

Therapy resistance in breast cancer is increasingly linked to polyploid giant cancer cells (PGCCs), which arise through whole genome doubling and exhibit heightened resistance to conventional treatments. To accelerate the discovery of PGCC-targeting compounds, we developed a high-throughput single-cell morphological analysis workflow that rapidly differentiates inhibitors of non-PGCCs, PGCCs, or both. Unlike flow cytometry, which struggles with cell dissociation, cluster removal, and dynamic tracking, our imaging-based approach is faster and more scalable and leverages computational advancements for superior screening efficiency. By screening 2726 FDA Phase 1-approved drugs, we identified promising anti-PGCC candidates, including inhibitors of the proteasome, FOXM1, CHK, and macrocyclic lactones. RNA-Seq analysis further implicated AXL inhibition as a potential PGCC-targeting strategy. To scale discovery, we developed an ensemble learning model integrating chemical fingerprints and compound descriptors to predict anti-PGCC efficacy. This model successfully predicted effective compounds from the PRISM library, which includes over 6000 drugs, with five top-ranked predictions experimentally validated. These findings highlight the power of AI-driven virtual screening in overcoming therapy resistance. With future data accumulation, our computational framework will continue to improve to enhance predictive accuracy and broader applicability in drug discovery.

## Supporting Information Available

Detailed methodologies for cell culture; image acquisition; whole-transcriptome sequencing; functional enrichment analysis of Pyronaridine treatment; and statistical analysis, along with Table S1 (PDF)
