# Supplementary material for: High-Throughput Empirical and Virtual Screening To Discover Novel Inhibitors of Polyploid Giant Cancer Cells in Breast Cancer
Source: Anal Chem. 2025 Mar 5;97(10):5498–506. doi: 10.1021/acs.analchem.4c05138 (PMC11923954; doi:10.1021/acs.analchem.4c05138)
Supplement: Supplementary file 1 — ac4c05138_si_001.pdf [file ac4c05138_si_001.pdf]

Supplementary Information

High-Throughput Empirical and Virtual Screening to Discover Novel Inhibitors of Polyploid Giant Cancer Cells in Breast Cancer

Yushu Ma, Chien-Hung Shih, Jinxiong Cheng, Hsiao-Chun Chen, Li-Ju Wang, Yanhao Tan, Yuan Zhang, Daniel D. Brown, Steffi Oesterreich, Adrian V. Lee, Yu-Chiao Chiu*, Yu-Chih Chen*

*Corresponding authors

Yu-Chih Chen, Ph.D.

5115 Centre Ave, Pittsburgh, PA 15232, USA

Tel: +1-412-623-7701; E-mail: cheny25@upmc.edu

Yu-Chiao Chiu, Ph.D.

5051 Centre Ave, Pittsburgh, PA 15231, USA

Tel: +1-412-648-5023; E-mail: YUC250@pitt.edu

- Supplementary Methods
- Supplementary References
- Supplementary Table S1

Supplementary Methods

Cell culture

We cultured MDA-MB-231, Vari068, and PDXO-073 cells in Dulbecco's Modified Eagle Medium (DMEM, Gibco 11995) supplemented with 10% fetal bovine serum (FBS, Gibco 16000), 1% GlutaMax (Gibco 35050), 1% penicillin/streptomycin (pen/strep, Gibco 15070), and 0.1% plasmocin (InvivoGen ant-mpp). SUM159 cells were cultured in F-12 medium (Gibco 11765) supplemented with 5% FBS (Gibco 16000), 1% pen/strep (Gibco 15070), 1% GlutaMax (Gibco 35050), 1 μg/mL hydrocortisone (Sigma H4001), 5 μg/mL insulin (Sigma I6634), and 0.1% Plasmocin (InvivoGen ant-mpp). MDA-MB-231 and SUM159 cells were obtained from Dr. Gary Luker's lab at the University of Michigan, Vari068 cells were obtained from Dr. Max Wicha's lab at the University of Michigan, and PDXO-073 cells were obtained from the Institute for Precision Medicine, University of Pittsburgh. The Vari068 cells, derived from an ER-/PR-/Her2- breast cancer patient who provided informed consent, were adapted to a standard two-dimensional culture environment.[1-3] The PDXO-073 cells were originally derived from a PDX (CTG-1260) that has an *ESR1* D538G mutation.[4] All cell cultures were maintained at 37 °C in a humidified incubator with 5% CO2 and passaged upon reaching over 80% confluency. All cell lines were cultured with a mycoplasma antibiotic Plasmocin.

Image acquisition

Cells in 96-well plates were imaged using an inverted Nikon Ti2E microscope. Brightfield and fluorescence images were captured with a 4x objective lens and a Hamamatsu ORCA-Fusion Gen-III SCMOS monochrome camera. Each field of view covers approximately 14 mm^2, accommodating up to 10,000 cells per image. Hoechst-stained cell nuclei were visualized with a DAPI filter set, while live and dead cells were detected using FITC and TRITC filter sets, respectively. Auto-focusing ensured image clarity, with the entire imaging process for a 96-well plate completed in under 9 minutes.

Whole-transcriptome sequencing

We extracted RNA from MDA-MB-231 cells, both untreated and treated with 1 μM Pyronaridine Tetraphosphate for 2 days, using the PureLink™ RNA Mini Kit (Invitrogen™, 12183018A). The RNA samples were processed at the UPMC Hillman Cancer Center Cancer Genomics Facility with a KAPA RNA HyperPrep Kit with RiboErase. Each sample population was expected to generate approximately 40 million reads (38x38 base paired-end), with two biological replicates conducted. Reads were aligned using Bowtie2 read aligners in Partek, followed by transcriptome assembly and differential expression analysis with DESeq2.[5, 6]

Functional enrichment analysis of the Pyronaridine treatment

Gene Set Enrichment Analysis (GSEA) was performed to understand the underlying mechanisms of Pyronaridine treatment.[7] Genes from RNA-seq were ranked based on the

statistical significance (*P*-value) of their differential expression in Pyronaridine-treated MDA-MB-231 cells compared to untreated cells. The curated gene sets representing genetic and chemical perturbations (CGPs) from the Molecular Signatures Database (MSigDB) were tested for enrichment at the negative end of the ranked gene list (*i.e.*, downregulated genes in response to Pyronaridine).[8] To analyze overlaps among enriched gene sets, we utilized EnrichmentMap and AutoAnnotate in Cytoscape for constructing and visualizing a gene set association network.[9] Gene set associations were represented by the degree of gene overlap between two sets, calculated as the average of the Jaccard index and the overlap coefficient (referred to as the combined coefficient). Gene sets with an FDR *q*-value below 0.05 in GSEA and a combined coefficient above 0.375 were included in the association network. Additionally, we analyzed the leading-edge subset of an enriched gene set of interest identified by GSEA, which represents the top-ranked genes that contribute most to the enrichment score. This subset was further studied for its potential relevance in the response to Pyronaridine.

Statistical analysis

Statistical analyses were conducted using R (version 4.1), GraphPad Prism 10, and MATLAB. GraphPad Prism 10 software determined half-maximal inhibitory concentrations (IC50s). Two-tailed Student's *t*-test compared two groups, while paired 1-way ANOVA and Fisher's Least Significant Difference (LSD) test compared multiple groups, considering treatment conditions as the variable. Within each cell line, treated versus untreated conditions were consistently paired for comparisons, with significance set at $P < 0.05$. The standard deviation was represented by error bars; sample/group details were specified in figure captions. For data with high variability (*e.g.*, gene expression levels), comparisons were made on a log scale.

Supplementary References

1. Liu, M.; Liu, Y.; Deng, L.; Wang, D.; He, X.; Zhou, L.; Wicha, M. S.; Bai, F.; Liu, S., Transcriptional profiles of different states of cancer stem cells in triple-negative breast cancer. *Mol Cancer* **2018,** *17* (1), 65.
2. Aw Yong, K. M.; Ulintz, P. J.; Caceres, S.; Cheng, X.; Bao, L.; Wu, Z.; Jiagge, E. M.; Merajver, S. D., Heterogeneity at the invasion front of triple negative breast cancer cells. *Sci Rep* **2020,** *10* (1), 5781.
3. Chen, Y. C.; Ingram, P. N.; Fouladdel, S.; McDermott, S. P.; Azizi, E.; Wicha, M. S.; Yoon, E., High-Throughput Single-Cell Derived Sphere Formation for Cancer Stem-Like Cell Identification and Analysis. *Sci Rep* **2016,** *6*, 27301.
4. Wu, Y.; Li, Z.; Wedn, A. M.; Casey, A. N.; Brown, D.; Rao, S. V.; Omarjee, S.; Hooda, J.; Carroll, J. S.; Gertz, J.; Atkinson, J. M.; Lee, A. V.; Oesterreich, S., FOXA1 Reprogramming Dictates Retinoid X Receptor Response in ESR1-Mutant Breast Cancer. *Mol Cancer Res* **2023,** *21* (6), 591-604.
5. Love, M. I.; Huber, W.; Anders, S., Moderated estimation of fold change and dispersion for RNA-seq data with DESeq2. *Genome Biol* **2014,** *15* (12), 550.
6. Langmead, B.; Salzberg, S. L., Fast gapped-read alignment with Bowtie 2. *Nat Methods* **2012,** *9* (4), 357-9.
7. Subramanian, A.; Tamayo, P.; Mootha, V. K.; Mukherjee, S.; Ebert, B. L.; Gillette, M. A.; Paulovich, A.; Pomeroy, S. L.; Golub, T. R.; Lander, E. S.; Mesirov, J. P., Gene set enrichment analysis: a knowledge-based approach for interpreting genome-wide expression profiles. *Proc Natl Acad Sci U S A* **2005,** *102* (43), 15545-50.
8. Liberzon, A.; Subramanian, A.; Pinchback, R.; Thorvaldsdottir, H.; Tamayo, P.; Mesirov, J. P., Molecular signatures database (MSigDB) 3.0. *Bioinformatics* **2011,** *27* (12), 1739-40.
9. Shannon, P.; Markiel, A.; Ozier, O.; Baliga, N. S.; Wang, J. T.; Ramage, D.; Amin, N.; Schwikowski, B.; Ideker, T., Cytoscape: a software environment for integrated models of biomolecular interaction networks. *Genome Res* **2003,** *13* (11), 2498-504.

Supplementary Table

Table S1. Predicted and validated drug responses for AV-412, Azeliragon, Lestaurtinib, Selamectin, and UCN-01 in two TNBC cell lines (MDA-MB-231 and SUM159) and two low-passage, patient-derived cells (Vari068 and PDXO-073).

	Prediction	Validation			
Drug	MDA-MB-231	MDA-MB-231	SUM159	Vari068	PDXO-073
AV-412	-3.1	-3.7	-0.6	-0.2	0.2
Azeliragon	-2.7	-4.2	-1.3	-0.6	-1.2
Lestaurtinib	-1.4	-1.4	-2.4	-1.0	-1.1
Selamectin	-5.9	-1.4	-0.8	-0.2	-0.2
UCN-01	-1.7	-2.3	-1.4	-1.2	-0.2
